# Prevention of gestational diabetes through lifestyle intervention: study design and methods of a Finnish randomized controlled multicenter trial (RADIEL)

**DOI:** 10.1186/1471-2393-14-70

**Published:** 2014-02-14

**Authors:** Kristiina Rönö, Beata Stach-Lempinen, Miira M Klemetti, Risto J Kaaja, Maritta Pöyhönen-Alho, Johan G Eriksson, Saila B Koivusalo

**Affiliations:** 1 Department of Obstetrics and Gynecology, University of Helsinki, Helsinki, Finland; 2 Department of Obstetrics and Gynecology, Helsinki University Central Hospital, Haartmaninkatu 2, P.O. Box 140, 00029 Helsinki, Finland; 3 Department of Obstetrics and Gynecology, South-Karelia Central Hospital, Lappeenranta, Finland; 4 Satakunta Central Hospital, Pori, Finland; 5 University of Turku, Turku, Finland; 6 Department of General Practice and Primary Health Care, University of Helsinki, Helsinki, Finland; 7 Unit of General Practice, Helsinki University Central Hospital, Helsinki, Finland; 8 Folkhälsan Research Centre, Helsinki, Helsingfors Universitet, Helsinki, Finland; 9 Department of Chronic Disease Prevention, National Institute for Health and Welfare, Helsinki, Finland

**Keywords:** Gestational diabetes, Type 2 diabetes, Diet and exercise intervention, Obesity, BMI, Pregnancy

## Abstract

**Background:**

Maternal overweight, obesity and consequently the incidence of gestational diabetes are increasing rapidly worldwide. The objective of the study was to assess the efficacy and cost-effectiveness of a combined diet and physical activity intervention implemented before, during and after pregnancy in a primary health care setting for preventing gestational diabetes, later type 2 diabetes and other metabolic consequences.

**Methods:**

RADIEL is a randomized controlled multi-center intervention trial in women at high risk for diabetes (a previous history of gestational diabetes or prepregnancy BMI ≥30 kg/m^2^). Participants planning pregnancy or in the first half of pregnancy were parallel-group randomized into an intervention arm which received lifestyle counseling and a control arm which received usual care given at their local antenatal clinics. All participants visited a study nurse every three months before and during pregnancy, and at 6 weeks, 6 and 12 months postpartum. Measurements and laboratory tests were performed on all participants with special focus on dietary and exercise habits and metabolic markers.

Of the 728 women [mean age 32.5 years (SD 4.7); median parity 1 (range 0-9)] considered to be eligible for the study 235 were non-pregnant and 493 pregnant [mean gestational age 13 (range 6 to 18) weeks] at the time of enrollment. The proportion of nulliparous women was 29.8% (n = 217). Out of all participants, 79.6% of the non-pregnant and 40.4% of the pregnant women had previous gestational diabetes and 20.4% of the non-pregnant and 59.6% of the pregnant women were recruited because of a prepregnancy BMI ≥30 kg/m^2^. Mean BMI at first visit was 30.1 kg/m^2^ (SD 6.2) in the non-pregnant and 32.7 kg/m^2^ (SD 5.6) in the pregnant group.

**Discussion:**

To our knowledge, this is the first randomized lifestyle intervention trial, which includes, besides the pregnancy period, both the prepregnancy and the postpartum period. This study design also provides an opportunity to focus upon the health of the next generation. The study is expected to produce novel information on the optimal timing and setting of interventions and for allocating resources to prevent obesity and diabetes in women of reproductive age.

**Trial registration:**

Clinicaltrials.gov Identifier: NCT01698385

## Background

Over the past few decades, obesity has become a global health challenge. In Finland, approximately every third parturient is overweight (body mass index (BMI) ≥ 25 kg/m^2^) and 13% are obese (BMI ≥ 30 kg/m^2^) [1]. The obesity epidemic among women of reproductive age has led to an increasing incidence of gestational diabetes (GDM) [1,2].

Gestational diabetes increases the likelihood of various perinatal complications, including fetal macrosomia [3-6]. Maternal obesity and GDM are independently associated with perinatal complications. The combined adverse effect of these two risk factors on the frequency of adverse obstetric outcomes is greater than that of either one alone [7-9]. A Swedish population-based study, which compared the time periods 1991-1997 and 1998-2008, reported no improvement in maternal and neonatal outcomes among GDM patients [5].

Up to 10% of women with previous GDM are diagnosed with type 2 diabetes soon after delivery. During a ten-year follow-up the risk may be as high as 70% [10]. Maternal obesity and hyperglycemia during pregnancy increase also the offspring’s risk of developing diabetes and obesity, promoting the intergenerational transmission of cardiometabolic disorders [11-14]. These observations underline the need for effective interventions that reduce obesity and prevent GDM among women of childbearing age.

Pregnant women may be particularly motivated to make healthy lifestyle changes. Recent data show that diet and exercise interventions may be successful in reducing gestational weight gain in women with an increased risk of developing GDM but their effects on the incidence of GDM and other adverse perinatal outcomes have been limited [15-20]. The sample sizes in most of these previous studies have been small. Furthermore, none of them included an intervention that started before pregnancy. Prepregnancy body size may be a stronger predictor for adverse obstetric and perinatal outcomes than weight gain during pregnancy [16,21]. Since pregnancy is a relatively brief period in life, diet and physical activity interventions should optimally be initiated already before pregnancy and continue after delivery to prevent the development of overt diabetes. To date, only a few postpartum intervention studies in women with previous GDM have been implemented with promising results in the reduction of cardiometabolic risk factors [22-24].

The Finnish Gestational Diabetes Prevention Study (RADIEL) is a randomized lifestyle intervention trial targeting women at high risk for diabetes when planning pregnancy or in the first half of pregnancy. The study was designed for a primary health care setting with the main aim to assess the efficacy and cost-effectiveness of a combined diet and physical activity intervention, implemented before, during and after pregnancy, in limiting gestational weight gain, preventing GDM and later type 2 diabetes, and reducing cardiovascular disease risk factors. The first phase of the study, including a 12-month follow-up postpartum, was completed in January 2014. In the second phase, subjects in the RADIEL cohort, including mothers, fathers and children, will be followed-up until the child is 10 years of age. This article presents the study design and methods of the first phase of the study as well as the baseline characteristics of the study population.

## Methods

### Study design and recruitment

The RADIEL-study is a multi-center randomized controlled intervention trial carried out between the years 2008 and 2014 in the maternity hospitals of the Helsinki metropolitan area (Helsinki University Central Hospital (HUCH) Department of Obstetrics and Gynecology, Kätilöopisto Maternity Hospital and Jorvi Hospital) and in the South-Karelia Central Hospital (SKCH) in Lappeenranta, in South-Eastern Finland.

Women with a previous history of GDM or a prepregnancy BMI ≥ 30 kg/m^2^, either planning pregnancy or pregnant at less than 20 + 0 weeks’ gestation, were eligible for the study. The subjects were recruited using newspaper and targeted social media notices, through primary health care centers and antenatal clinics as well as by personal invitation letters sent out based on hospital registry.

Exclusion criteria were: age <18 years, diabetes diagnosed before pregnancy, medications that influence glucose metabolism (e.g. oral corticosteroids and metformin), multiple pregnancies, physical disability, current substance abuse, severe psychiatric disorders and significant difficulties to co-operate (e.g. inadequate Finnish language skills). Miscarriage or fetal death after 22 + 0 weeks’ gestation was not a dropout criterion but an outcome of pregnancy and the mother was allowed to continue the follow-up.

The participants were randomized as described later into the intervention arm or the control arm of the study. All participants entered into the study voluntarily, signed an informed consent form and were allowed to discontinue at any point. The study complies with the Declaration of Helsinki [25] and was approved by Ethical Boards of HUCH (14 September 2006, Dnro 300/E9/06) and SKCH (11 September 2008, Dnro M06/08). The protocol was registered at clinicaltrials.gov (IDr: NCT01698385).

### Sample size and randomization

Sample size was calculated assuming a GDM incidence of 30% in the control arm and 20% incidence in the intervention arm. Using a two-sided significance level of 0.05 and a power of 80%, and assuming a dropout rate of 30%, a sample size of 1000 subjects was estimated to be sufficient. The sample size calculation was carried out with NQuery Advisor (6.0) using a continuity corrected chi-square test. Randomization was performed using randomly permuted blocks. Each subject was randomized by dispensing the next sequentially numbered subject code and opening the corresponding code envelope indicating the study arm to be assigned to the participant in question. The intervention and control arms were randomized in a balanced fashion separately within each study site.

### Intervention

The participants visited the study nurse in the hospital outpatient clinic every three months before and during pregnancy, and at 6 weeks, 6 months and 12 months postpartum.

In the intervention arm, the visits included structured counseling on diet and exercise (see below). Counseling was given by study nurses and nutritionists specifically trained for their tasks. The following weight targets were set: 5-10% weight loss before pregnancy was recommended for women with a prepregnancy BMI ≥ 25 kg/m^2^ and no weight gain during the first two trimesters of pregnancy was recommended for women with a prepregnancy BMI ≥ 30 kg/m^2^.

Participants in the control arm received basic dietary and exercise information leaflets similar to those provided at primary health care centers at the time of enrollment. During pregnancy, they received usual health education provided at their local antenatal clinic.

### Dietary intervention

Dietary counseling was based on the national Finnish nutritional guidelines [26,27]. "The plate model" used during the counseling sessions refers to filling half a plate with raw or cooked vegetables, one quarter with starchy carbohydrates (e.g. potato, rice or pasta) and one quarter with meat, fish, beans, eggs or other sources of protein. The aim was to achieve a total energy (E) intake of 1600-1800 kcal a day, with 40-50% of total energy (E%) coming from carbohydrates, 30-40 E% from fats and 20-25 E% from protein. The participants in the intervention arm were encouraged to increase their intake of vegetables, legumes, fruits and berries; whole grain and fiber; low-fat dairy and vegetable fats. During the postpartum period, breastfeeding and infant nutrition counseling were also provided based on national recommendations [26]. Every three months throughout the study, the participants filled in three-day food diaries, which were used both as motivational and educational tools and as data collection instruments.

In addition to regular visit to the study nurse participants took part in structured group visits to a nutritionist at the moment of enrollment in the study, during the first trimester of pregnancy as well as at 6 and 12 months postpartum. Additional individual visits were arranged when needed e.g. in case the dietary or weight management goals were not met or the study subject had special dietary restrictions.

### Exercise intervention

The aim of the physical activity counseling was to achieve a minimum of 30 minutes of moderate intensity exercise five times a week or 50 minutes three times a week and to adopt an overall active lifestyle including daily household physical activity and/or transportation physical activity. Moderate intensity exercise was defined as exercise during which the participant becomes at least slightly out of breath and perspires but is still able to talk or a level equaling 11-15 on Borg’s visual scale of perceived exertion [28,29]. An individual exercise program was planned for each participant during the counseling visits. The program was modified during the follow-up when needed. Participants received pedometers as motivational tools. The recommendation was a minimum of 10 000 steps a day. The participants had possibility to attend guided exercise groups provided by the municipalities or got ticket to e.g. public swimming pools once a week free of charge. In case exercise goals were not met, the study subjects were instructed to book an appointment with the physical activity advisor. The services by the municipal physical activity advisors were provided free of charge to everyone who wanted to receive extra counseling on exercise. Physical activity logbooks were used both as motivational and educational tools and as data collection instruments.

### Outcomes

The incidence of GDM was the primary outcome of the RADIEL study. GDM was defined as one or more pathological glucose value in a 75 g two-hour oral glucose tolerance test (OGTT) during pregnancy. The following diagnostic thresholds were applied: fasting plasma glucose ≥ 5.3 mmol/l, one hour value ≥ 10.0 mmol/l and two hour value ≥ 8.6 mmol/l [30]. Dietary treatment was initiated in the primary health care center immediately after diagnosis of GDM. In case glucose values in home measurements exceeded repeatedly 5.5 mmol/l before breakfast or 7.8 mmol/l one hour after a meal, insulin treatment was initiated [31].

Secondary outcomes were gestational weight gain and maternal body mass index (BMI), insulin sensitivity, achievement of dietary and physical activity goals, incidence of preeclampsia, incidence of gestational hypertension, mode of delivery, perinatal outcome, maternal quality of life, cost-effectiveness of the intervention in prevention of GDM and incidence of maternal type 2 diabetes one year after delivery. Preeclampsia was defined according to the criteria of the American College of Obstetricians and Gynecologists (ACOG) i.e. systolic blood pressure of ≥ 140 mm Hg or diastolic blood pressure of ≥ 90 mm Hg occurring after 20 weeks of gestation in a previously normotensive woman combined with new-onset proteinuria of ≥0.3 g/24 h [32,33]. Gestational hypertension was defined similarly but without the presence of proteinuria. Macrosomia was defined as birth weight > 2.0 SD and small for gestational age as birth weight < -2.0 SD using a Finnish standards adjusted for sex and gestational age [34]. Neonatal hypoglycaemia was defined as blood glucose <2.6 mmol/l in the first 48 hours of life.

The obstetric and perinatal records of each subject were reviewed and maternal and neonatal diagnoses confirmed by research physicians before analysis of endpoints was initiated.

### Data collection

Height, weight, waist and hip circumference (if not pregnant), resting blood pressure and heart rate were measured at the time of enrollment in the study. Self-reported prepregnancy weight was collected from maternity care cards. Height and weight were measured in light indoor clothing and without shoes on. Height was measured to the nearest 0.5 cm and weight to the nearest 0.1 kg. Blood pressure was measured from the right arm while the subject was in the sitting position using a sphygmomanometer.

Body mass index (BMI) was calculated as weight in kilograms divided by height in meters squared (kg/m^2^). Waist-hip-ratio was defined as waist circumference measured 2 cm above the umbilical level divided by the hip circumference measured at the widest portion of the buttocks. The same measurements were made at every follow-up visit except for the waist and hip measurements, which were not taken during pregnancy. Laboratory tests performed in conjunction with the visits included a 75 g 2-hour oral glucose tolerance test (OGTT), measurements of fasting plasma glucose and insulin, glycated hemoglobin (GHbA_1c_), total cholesterol, low-density lipoprotein (LDL) and high-density lipoprotein (HDL) cholesterol and triglycerides, alanine transaminase (ALAT), thyroid-stimulating hormone (TSH), free thyroxin (T_4_) and high-sensitive C-reactive protein (hsCRP) (Table 1). Blood samples for DNA extraction and further genetic analyses were drawn. In case the result of any laboratory test or other measurement performed as part of the RADIEL trial follow-up was abnormal, the participants were referred to the primary health care centers.

**Table 1:** Data collection in The Finnish Gestational Diabetes Prevention Study (RADIEL)

	**Enrollment**	**Prepregnancy follow-up every 3 months**	**1st trimester**	**2nd trimester**	**3rd trimester**	**Birth**	**6 weeks after delivery**	**6 months after delivery**	**12 months after delivery**
Questionnaires									
Background- and progress questionnaires									
Socioeconomic status	X						X		
Medical and obstetric history	X								
Self reported morbidity and use of medication	X	X	X	X	X		X	X	X
Smoking and alcohol consumption	X	X	X	X	X		X	X	X
Quality of life	X	X	X	X	X		X	X	X
Diet	X	X	X	X	X		X	X	X
Physical activity	X	X	X	X	X		X	X	X
EPDS	X		X				X	X	X
15D	X		X	X	X		X	X	X
Logbooks for food and physical activity	X		X		X				X
Physical measurements									
Height	X								
Weight and BMI	X	X	X	X	X		X	X	X
Waist-hip ratio	X	X					X	X	X
Resting blood pressure and heart rate	X	X	X	X	X		X	X	X
Laboratory tests									
75 g 2-hour OGTT	X		X	X			X		X
Glucose, insulin, GHbA_1c_ and hsCRP	X	X	X	X	X		X	X	X
ALAT, lipids	X						X		X
TSH and free T4	X						X		
DNA	X								
Spare blood for further analyses	X	X	X	X	X		X	X	X
Offspring									
Birth weight and length						X			
Apgar score						X			
Cord-blood gas analysis (pH)						X			
Cord-blood sample (DNA)						X			

EPDS = Edinburgh Postnatal Depression Scale; BMI = body mass index; OGTT = oral glucose tolerance test; GHbA_1c_ = glycated hemoglobin; hsCRP = high sensivity C-reactive protein; ALAT = alanin transaminase; TSH = thyroid-stimulating hormone; T4 = thyroxin.

Data on mode of delivery, blood loss during delivery, duration of labor, perineal tears, gestational age at birth, birth weight and length, umbilical artery pH and acid-base values, Apgar scores, lowest blood glucose values of the newborn infant, perinatal complications and neonatal intensive care unit admission were obtained from patient records. In addition, a sample of cord blood was collected at birth.

Information on socioeconomic status, self-reported morbidity, use of medication, perceived health status, quality of life, family history of diabetes and hypertension, obstetric history, smoking, consumption of alcohol, history of weight management and diet and exercise habits during the past six months were collected with a background questionnaire and an interview by a study nurse. Before every follow-up visit participants completed a questionnaire assessing changes in diet and exercise habits after the previous visit. After delivery, the questionnaires included also questions concerning delivery and breastfeeding. The 15D Questionnaire [35] for the assessment of health-related quality of life (HRQoL) and quality-adjusted life years (QALY) was completed at the enrollment visit as well as during pregnancy and post-partum follow up visits. A modified Edinburgh Postnatal Depression Scale (EPDS) questionnaire [36] for the assessment of depressive symptoms was used at the moment of enrollment, on the first trimester visit and on each visit after delivery.

Logbooks of food intake and physical activity were collected at enrollment, during the first and third trimester visits and one year after delivery. Three-day food diaries were filled for two weekdays and one day during the weekend before each visit. Physical activity logbooks, including records on physical activity type, frequency and intensity on Borg scale and pedometer readings (intervention group), were filled daily for one week at each time. Participants in the intervention group were encouraged to use logbooks as motivational tool between the visits.

### Statistical analysis

Means, range and/or standard deviation (SD) were used to describe continuous normally distributed data. Skew continuous data were described using median and range. Differences in means between the independent groups were tested using Student’s t-test and in medians using Mann-Whitney u-test. Cross-tabulated data was analyzed with Pearson’s chi-square test. The limit for significance was set equal to 0.05. Data analysis was carried out using SPSS for Windows (Version 21) and Mac (Version 20).

## Results

**Figure 1:**
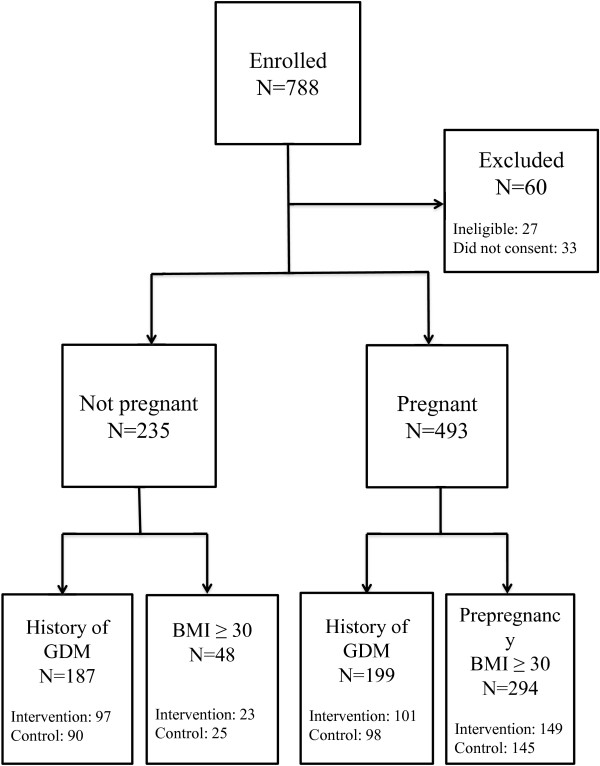
**Flow chart of participant recruitment.** GDM = gestational diabetes, BMI = body mass index.

Between February 2008 and November 2011, 788 women were recruited into the study. Sixty women were excluded: 27 did not meet the inclusion criteria and 33 did not provide a written informed consent. Of the 728 eligible women, 235 were non-pregnant and 493 pregnant during the first study visit. In the non-pregnant and pregnant groups, 79.6% (n = 187) and 40.4% (n = 199), respectively, had a history of previous GDM. Pre-pregnancy BMI ≥30 kg/m^2^ without a history of previous GDM was the recruitment criterion for 20.4% (n = 48) of women in the non-pregnant group and for 59.6% (n = 294) in the pregnant group (Figure 1). The history of GDM was verified by hospital records in 98.4% of the cases (n = 380). In six women, previous GDM was self-reported. Women with a history of previous GDM were on average leaner than women recruited only because of their BMI being ≥30 kg/m^2^ (Table 2). Of the participants with a history of GDM 35.3% (n = 66) in the non-pregnant and 42.7% (n = 85) in the pregnant group were obese.

**Table 2:** Baseline mean BMI and history of gestational diabetes by study group

	**Non-pregnant group**		Pregnant group*		**p Value**^**1 **^**Difference between the groups**
	**N**	**BMI mean (SD)**^**2**^	**N**	**BMI mean (SD)**^**2**^	
Previous GDM	187	28.6 (5.7)	199	29.4 (6.2)	0.19
No GDM history	48	36.0 (4.4)	291	35.0 (3.9)	0.11

BMI = body mass index; GDM = gestational diabetes.

*Mean weeks of gestation 13 (range 6 to 18).

^1^*P*-value based on Student’s t test.

^2^*P*-value based on Student’s t test on difference between participants with and without previous GDM <0.001.

Baseline characteristics of the study groups are presented in Tables 3, 4 and 5. There were more nulliparous women in the pregnant group (38.3%, n = 189) than in the non-pregnant group (11.9%, n = 28) (p < 0.001). In the non-pregnant group, 17.4% (n = 20) of women in the control arm and 6.7% (n = 8) in the intervention arm were nulliparous (p = 0.01). There were no other significant differences in any of the tested variables between the intervention and the control arm. 28.6% of the women (n = 208) reported some chronic disease, no significant difference was observed between non-pregnant and pregnant participants.

**Table 3:** Demographic and socioeconomic characteristics by study group

	**Study population**	**Non-pregnant group**	**Pregnant group**	***p-*** **Value**
				**Difference between groups**
	**N = 728**	**N = 235**	**N = 493**	
Age, Mean (SD) years	32.5 (4.7)	32.8 (4.2)	32.4 (4.9)	0.27^1^
Marital status, n (%)	N = 722	N = 234	N = 487	
Married/co-habiting	699 (96.8%)	230 (98.3%)	469 (96.1%)	
Other	23 (3.2%)	4 (1.7%)	19 (3.9%)	
				0.12^2^
Basic education, n (%)	N = 720	N = 234	N = 486	
Elementary school	165 (22.9%)	42 (17.9%)	123 (25.3%)	
Part of high school	31 (4.3%)	15 (6.4%)	16 (3.3%)	
High school diploma	514 (71.4%)	173 (73.9%)	341 (70.2%)	
Other	10 (1.4%)	4 (1.7%)	6 (1.2%)	
				0.049^2^
Vocational education, n (%)	N = 718	N = 232	N = 486	
No professional education/ diploma/degree	76 (10.6%)	25 (10.8%)	51 (10.5%)	
Vocational course/school or apprenticeship	236 (32.9%)	70 (30.2%)	166 (34.2%)	
Vocational diploma/degree	174 (24.2%)	54 (23.3%)	120 (24.7%)	
Academic degree	228 (31.8%)	83 (35.8%)	145 (29.8%)	
Other	4 (0.6%)	0 (0.0%)	4 (0.8%)	
				0.34^2^
Current work situation, n (%)	N = 722	N = 234	N = 488	
Full-time work	368 (51.0%)	75 (32.1%)	293 (60.0%)	
Part-time work	70 (9.7%)	25 (10.7%)	45 (9.2%)	
Housewife	191 (26.5%)	87 (37.2%)	104 (21.3%)	
Unemployed	16 (2.2%)	4 (1.7%)	12 (2.5%)	
Maternity leave	36 (5.0%)	29 (12.4%)	7 (1.4%)	
Other	41 (5.7%)	14 (6.0%)	27 (5.5%)	
				<0.001^2^
Annual household income, n (%)	N = 689	N = 226	N = 463	
<20 000 euro/year	33 (4.8%)	9 (4.0%)	24 (5.2%)	
20 001-50 000	251 (36.4%)	83 (36.7%)	168 (36.3%)	
50 001-100 000	366 (53.1%)	116 (51.3%)	250 (54.0%)	
>100 000	39 (5.7%)	18 (8.0%)	21 (4.5%)	
				0.28^2^

^1^*P*-value based on Student’s t test.

^2^*P*-values based on Pearson’s χ^2^ test.

**Table 4:** Baseline health characteristics by study group

	**Study population**	**Non-pregnant group**	**Pregnant group**	**p value** **Difference between groups**
Smoking status, n (%)	N = 728	N = 235	N = 493	
Non-smoker	678 (93.1%)	215 (91.5%)	463 (93.9%)	
Smoking regularly	46 (6.3%)	18 (7.7%)	28 (5.7%)	
Smoking occasionally	4 (0.5%)	2 (0.9%)	2 (0.4%)	
				0.44^1^
Alcohol consumption, n (%)	N = 719	N = 232	N = 487	
None (0 portions a week)	567 (78.9%)	100 (43.1%)	467 (95.9%)	
Less than 5 portions a week	143 (19.9%)	123 (53.0%)	20 (4.1%)	
5-10 portions a week	7 (1.0%)	7 (3.0%)	0 (0.0%)	
More than 10 but not more than 16 portions a week	2 (0.3%)	2 (0.9%)	0 (0.0%)	
				<0.001^1^
BMI (kg/m^2)^	N = 725	N = 235	N = 490	
Mean (SD)	31.9 (6.0)	30.1 (6.2)	32.7 (5.6)	<0.001^2^
				
BMI grouping, n (%)	N = 725	N = 235	N = 490	
Underweight (< 18,5)	4 (0.6%)	2 (0.9%)	2 (0.4%)	
Normal (18,5-24,9)	105 (14.5%)	57 (24.3%)	48 (9.8%)	
Overweight (25,0-29,9)	132 (18.2%)	62 (26.4%)	70 (14.3%)	
Moderately obese (30,0-34,9)	270 (37.2%)	62 (26.4%)	208 (42.4%)	
Severely obese (35,0-39,9)	160 (22.1%)	36 (15.3%)	124 (25.3%)	
Very severely obese (≥ 40,0 or more)	54 (7.4%)	16 (6.8%)	38 (7.8%)	
				<0.001^1^
Pre-pregnancy BMI (kg/m^2^)			N = 492	
Mean (SD)			32.2 (5.8)	
				
Pre-pregnancy BMI grouping n (%)			N = 492	
Underweight (less than 18,5)			2 (0.4%)	
Normal (18,5-24,9)			66 (13.4%)	
Overweight (25,0-29,9)			59 (12.0%)	
Moderately obese (30,0-34,9)			222 (45.1%)	
Severely obese (35,0-39,9)			107 (21.7%)	
Very severely obese (40,0 or more)			36 (7.3%)	
				
Parental history of diabetes or cardiovascular disease, n (%)	N = 705	N = 229	N = 476	
No	263 (37.3%)	82 (35.8%)	181 (38.0%)	
Yes	442 (62.7%)	147 (64.2%)	295 (62.0%)	
Type I diabetes	10 (1.4%)	2 (0.9%)	8 (1.7%)	0.40^1^
Type II diabetes	197 (27.9%)	69 (30.1%)	128 (26.9%)	0.37^1^
Hypertension	351 (49.8%)	114 (49.8%)	237 (49.8%)	1.00^1^
Coronary artery disease	88 (12.5%)	30 (13.1%)	58 (12.2%)	0.73^1^
				
	N = 714	N = 232	N = 482	
Parental history of overweight or obesity, n (%)	527 (73.8%)	172 (74.1%)	355 (73.7%)	0.89^1^
				
Maternal overweight or obesity, n (%)	N = 702	N = 229	N = 473	
Yes	386 (55.0%)	123 (53.7%)	263 (55.6%)	
Yes, before, but not any more	1 (0.1%)	1 (0.4%)	0 (0.0%)	
No	315 (44.9%)	105 (45.9%)	210 (44.4%)	0.33^1^
				
Paternal overweight or obesity, n (%)	N = 697	N = 225	N = 472	
Yes	332 (47.6%)	114 (50.7%)	218 (46.2%)	
Yes, before, but not any more	9 (1.3%)	0 (0.0%)	9 (1.9%)	
No	356 (51.1%)	111 (49.3%)	245 (51.9%)	0.08^1^

BMI = body mass index.

^1^*P*-values based on Pearson’s χ^2^ test.

^2^*P*-value based on Student’s t test.

^3^*p* value based on Mann-Whitney *U test.*

**Table 5:** Participants’ pregnancy history by study group

	**Study population**	**Non-pregnant group**	**Pregnant group**	***p *** **Value**
				**Difference between groups**
	**N = 728**	**N = 235**	**N = 493**	
Median numbers of pregnancies (min-max)	1 (0-12)	1 (0-8)	1 (0-12)	0.003^1^
Parity median (min-max)	1 (0-9)	1 (0-7)	1 (0-9)	
History of pregnancy complications, n (%)	N = 511	N = 207	N = 304	
History of gestational diabetes	38 (75,5%)	187 (90,3%)	199 (65,5%)	<0,001^3^
Gestational diabetes treated with medication	56 (11,0%)	26 (12,6%)	30 (9,9%)	0,34^3^
History of preeclampsia	43 (8,4%)	13 (6,3%)	30 (9,9%)	0,15^3^
History of caesarian section	157 (30,7%)	64 (30,9%)	93 (30,6%)	0,94^3^
History of preterm birth	23 (4,5%)	11 (5,3%)	12 (3,9%)	0,46^3^
Birth weight of children, n (%)	N = 500	N = 205	N = 295	
Mothers with macrosomic babies (4500 g or more)	42 (8,4%)	14 (6,8%)	28 (9,5%)	0,29^3^
Mothers with small babies (less than 2500 g)	31 (6,2%)	11 (5,4%)	20 (6,8%)	0,52^3^

^1^*p* value based on Mann-Whitney *U test.*

^*2*^*p* value based on Student’s t-test.

^3^*p* value based on Pearson’s χ^2^ test.

In the pregnant group, the mean gestational age was 13 + 1 (range 5 + 5 to 18 + 3) weeks at the first visit to the study nurse. 3.1% of these pregnancies (n = 15) were initiated with an embryo transfer. Participants who had had an embryo transfer underwent the same study protocol as the rest of the study population.

## Discussion

The prevalence of overweight and obesity among women of childbearing age is rapidly increasing. This is leading to an increasing incidence of gestational diabetes and other metabolic and obstetric complications. This rising problem has led to a growing interest within the scientific community to identify effective intervention measures in order to interrupt the vicious cycle of obesity and adverse health outcomes among pregnant women. Successful weight loss in high risk obese women is known to have large impact on later risk of type 2 diabetes and cardiovascular diseases [37]. Weight control during pregnancy may also decrease the risk of obesity in long term [22,38]. However lifestyle changes are difficult to achieve and maintain due to both physiological and behavioral factors. There are several reasons to believe that a timely intervention during an early, plastic phase of fetal development may also lead to improved lifelong health of the newborn [39-42].

To our knowledge, the RADIEL study is the first randomized GDM prevention study that includes both the prepregnancy and postpartum period. Intervention during prepregnancy provides an opportunity to promote healthy maternal dietary habits and weight management before conception and during the early phases of pregnancy. This might be important in terms of fetal programming. The post-partum intervention might be significant in terms of improving the next generation’s health and nutrition. Most women in our study had a family history of overweight or obesity and almost a third had a family history of type 2 diabetes, which show that they do belong to a high risk group and furthermore underline the need to target the whole family when aiming at diabetes prevention. Gestational weight gain is determined by a multitude of factors [43] of which maternal diet and physical activity are considered to be the most modifiable. This was also the hypothesis when designing the RADIEL study. Diet and exercise interventions are generally considered to be safe in pregnant women [15,16]. In order to further improve the safety of the intervention, the study nurses were midwives with strong expertise in counseling pregnant women. Lifestyle intervention methods have previously been successfully implemented in the prevention of type 2 diabetes in non-pregnant individuals at increased risk for type 2 diabetes [37].

When the RADIEL study was planned, no previous studies in this field existed. Taking into account the limited information available, it was decided to collect a wide range of phenotypic data at baseline but also during the intervention in order to establish predictors of successful intervention as well as modifying factors. The study included women at high risk of developing GDM and therefore the RADIEL cohort is not representative of all pregnant Finnish women. Furthermore, like in most randomized controlled studies applying life style intervention, the control group is not a true control group since they also received general information about a healthy lifestyle. This might affect the overall results.

In our study population, there are significantly more nulliparous women in the pregnant group than in the non-pregnant group. This was the result of the recruitment methods used. Women in the non-pregnant group were recruited mainly by personal invitation letters based on hospital registry of previous GDM diagnosis. Women in the pregnant group came to the study mainly from antenatal clinics and two thirds of them were recruited due to their high BMI. Our study subjects are mainly non-smokers, most of them well educated, with on average quite good annual household income [44].

Obese women planning pregnancy, especially nulliparous, proved to be difficult to recruit, despite extensive and repeated recruitment efforts and a long recruitment time. Therefore the planned total sample size was not met in non-pregnant group. However, the number of women recruited should be sufficient to show whether the intervention initiated already in prepregnancy is more efficient than an intervention initiated in early pregnancy.

The RADIEL study focuses upon GDM and the impact of a lifestyle intervention from several different angles: perinatology, paediatrics, diabetology and neuroendocrinology, exercise medicine, genetics, nutrition and health economics. The mother and child cohort creates a unique material, which can be followed up for years to examine the long-term effects of the intervention. The study is expected to produce novel information that can easily be applied in primary heath care systems when selecting interventions and allocating resources to prevent obesity and diabetes in women of reproductive age.

## Competing interests

The authors declare that they have no competing interests.

## Authors’ contributions

SK initiated the study, participated in the design of the study, coordinated the study and helped in drafting and editing of the manuscript. KR participated in the implementation of the study, prepared the database, performed the statistical analyses and participated in the drafting of the manuscript. BSL participated in the design of the study, coordinated the study in Lappeenranta and helped with the statistical analyses and drafting of the manuscript. MK participated in the design and implementation of the study and the drafting of the manuscript*.* RK and MPA participated in the design of the study. JE is the principal investigator of the study and advised on editing the manuscript. All authors have read and approved the final version of the manuscript.

## Authors’ information

The members of the RADIEL group are: Carita Ahmala, Maaret Ahola, Kirsi Arponen, Sture Andersson, Johan Eriksson, Pirkko Haapanen, Karoliina Himanen, Emilia Huvinen, Risto Kaaja, Miira Klemetti, Saila Koivusalo, Päivi Kylliäinen, Hannele Laivuori, Johanna Metsälä, Hanna Oksa, Maritta Pöyhönen-Alho, Risto Roine, Kristiina Rönö, Niina Sahrakorpi and Aila Tiitinen.

## Pre-publication history

The pre-publication history for this paper can be accessed here:

http://www.biomedcentral.com/1471-2393/14/70/prepub
